# Luminescence Thermometry for Brain Activity Monitoring: A Perspective

**DOI:** 10.3389/fchem.2022.941861

**Published:** 2022-07-12

**Authors:** Paloma Rodríguez-Sevilla, Riccardo Marin, Erving Ximendes, Blanca del Rosal, Antonio Benayas, Daniel Jaque

**Affiliations:** ^1^ Departamento de Física de Materiales, Nanomaterials for Bioimaging Group (NanoBIG), Facultad de Ciencias, Universidad Autónoma de Madrid, Madrid, Spain; ^2^ Nanomaterials for Bioimaging Group (NanoBIG), Instituto Ramón y Cajal de Investigación Sanitaria (IRYCIS), Hospital Ramón y Cajal, Madrid, Spain; ^3^ School of Science, RMIT University, Melbourne, VIC, Australia

**Keywords:** luminescence thermometry, brain diagnosis, *in vivo*, thermal diagnosis, brain activity

## Abstract

Minimally invasive monitoring of brain activity is essential not only to gain understanding on the working principles of the brain, but also for the development of new diagnostic tools. In this perspective we describe how brain thermometry could be an alternative to conventional methods (e.g., magnetic resonance or nuclear medicine) for the acquisition of thermal images of the brain with enough spatial and temperature resolution to track brain activity in minimally perturbed animals. We focus on the latest advances in transcranial luminescence thermometry introducing a critical discussion on its advantages and shortcomings. We also anticipate the main challenges that the application of luminescent nanoparticles for brain thermometry will face in next years. With this work we aim to promote the development of near infrared luminescence for brain activity monitoring, which could also benefit other research areas dealing with the brain and its illnesses.

## Measuring Brain Activity in Freely Moving Animals: Motivation and State of the Art

The brain controls the vital functions and determines the way external stimuli are processed and interpreted. Diseases affecting the brain are a major contributor to mortality worldwide and reduce drastically the quality of life of the affected patients, imposing a great burden on healthcare services. (Olesen and Leonardi, 2003; Olesen et al., 2012). Existing therapies generally aim to treat the symptoms rather than address the cause. Despite the unquestionable relevance of brain dysfunctions, they are not completely understood, hence hindering the development of adequate treatments. Preclinical research, which involves designing and developing experiments in small animal models, is essential towards an in-depth understanding of brain function. Most of these studies focus on continuous recording of brain activity, which provides a direct indication of the presence of dysfunctions. Nowadays, preclinical imaging of brain activity is mainly achieved thorough two technologies:

- *Functional Magnetic Resonance Imaging* (fMRI). This technique provides high-resolution images of brain activity by detecting changes associated with blood flow. fMRI is based on the relation between local blood flow and neuronal activation.
- *Positron Emission Tomography* (PET). This method identifies alterations of brain activity by localizing areas with elevated metabolic activity. PET uses radioactive tracers usually attached to glucose-based compounds that accumulate in brain compartments with higher metabolic activity (larger glucose consumption).

**FIGURE 1:**
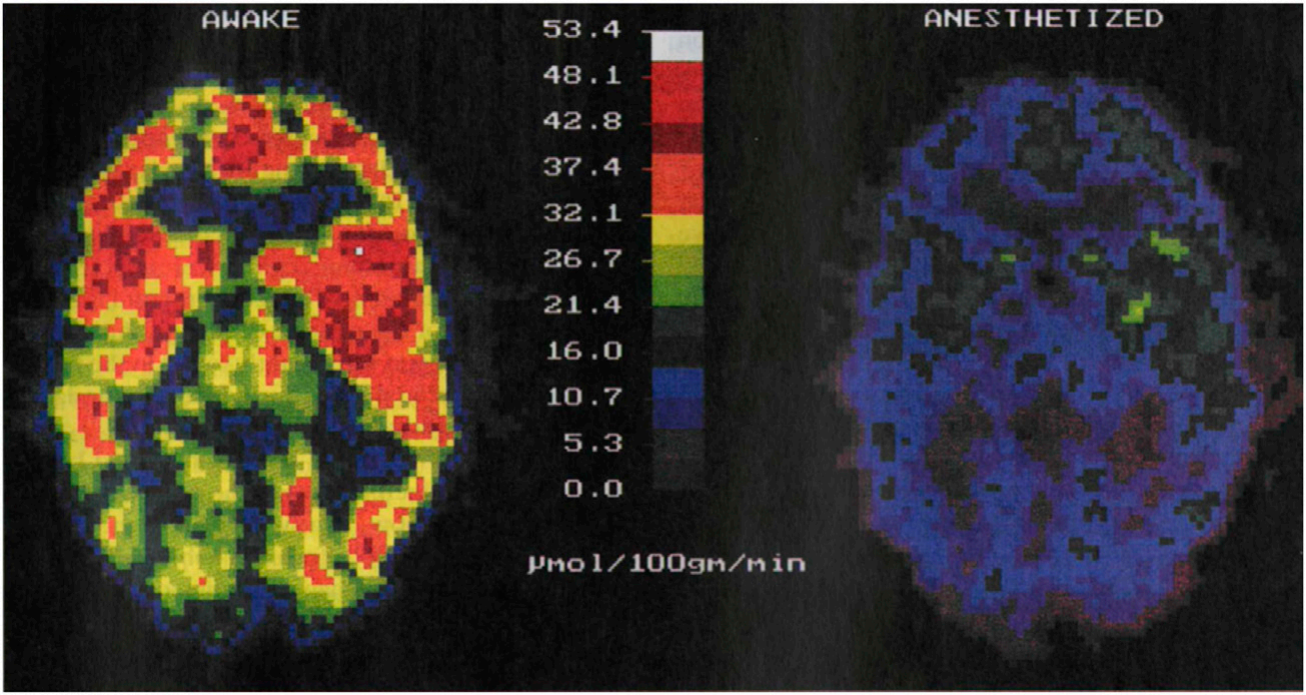
PET images of brain activity (i.e., glucose metabolism in µmol/100 g/min) obtained from the same individual awake, left, and under anaesthesia, right. The activity of the brain decreases in anesthetized patients. Image reproduced with permission from Reference (Alkire et al., 1995).

Despite their widespread use, each of them has its own limitations. fMRI requires expensive MRI systems, whereas PET uses ionizing radiation. In addition, they entail a severe alteration of the normal life conditions of the subject under study as they require its complete immobilization by mechanical constraints (stressful procedure) or the use of anaesthesia. Previous results have demonstrated that both stress and anaesthesia lead to significant alterations in the activity of the brain: whereas stress causes an increment in brain activity, anaesthesia strongly suppresses it (Alkire et al., 1995; Heinke and Koelsch, 2005; Sung et al., 2009; Boveroux et al., 2010). Indeed, PET imaging experiments have revealed a 57% reduction of brain activity under anaesthesia (Figure 1). Immobilization-induced stress has been found to cause more complex changes in brain activity: while brain areas such as the hypothalamus, entorhinal, and insular/piriform cortices were activated by immobilization stress, many more regions were deactivated. (Sung et al., 2009).

Thus, both anaesthesia and immobilization significantly alter the monitored parameter—brain activity—and, hence, hamper the possibility to study of the brain while it is working in normal conditions. In other words, the definitive understanding of how the brain works should avoid experiments on immobilized and/or anaesthetized specimens, since in those situations the functioning of their brains is altered/diminished. Therefore, brain activity should be monitored on awake and freely-moving animals by using contactless methods.

**FIGURE 2:**
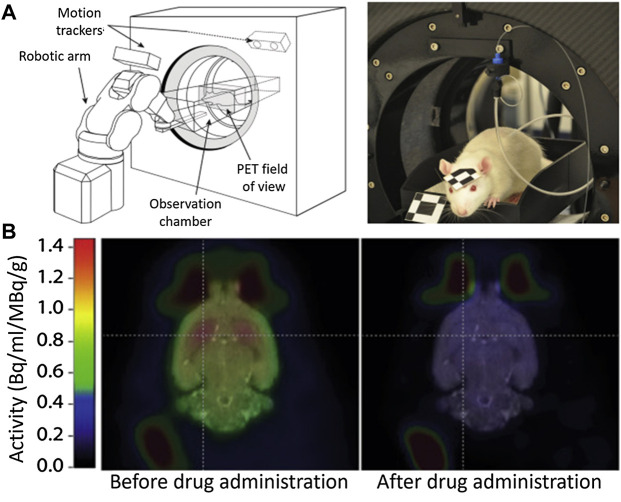
**(A)** Experimental setup to measure brain activity in non-immobilized mice. **(B)** PET Images of the cerebral activity obtained from an awake rat before and after drug (amphetamine) administration. Published with permission from Reference (Kyme et al., 2019).

This need has motivated the community to upgrade the traditional techniques. Very recently, Kyme and co-workers developed an experimental setup capable of acquiring PET images in a freely moving and awake rat (Kyme et al., 2019). The experimental system (Figure 2A) consisted of an unmodified small animal PET system, a robot-controlled animal enclosure, and an optical motion-tracking device (Kyme et al., 2011; Kyme et al., 2019). Using this system, the authors were capable to image the impact of amphetamine on the brain activity without the need of immobilization (Figure 2B). Despite the important results they obtained, the system still implies severe motion restrictions and makes use of ionizing radiation.

**FIGURE 3:**
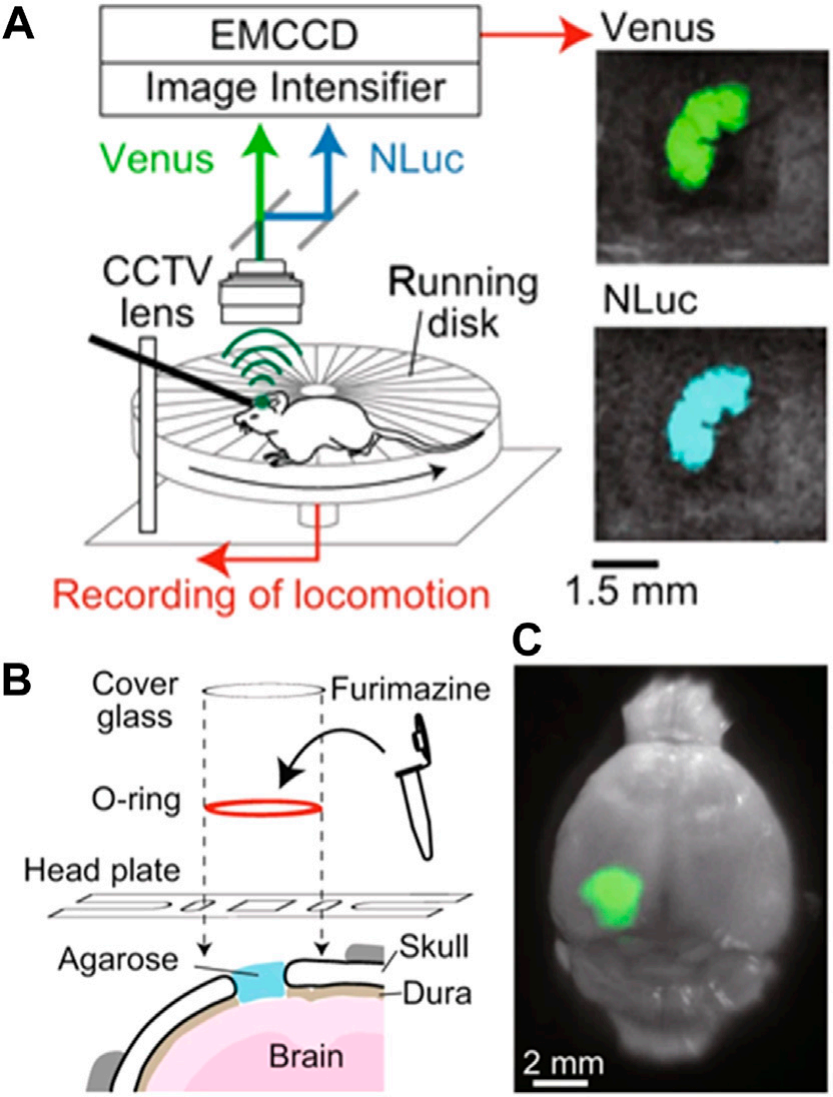
**(A)** Schematic representation of the experimental setup developed to monitor brain activity in freely-moving mice by using a ratiometric fluorescent probe. **(B)** Schematic representation of the procedure followed to insert the ratiometric fluorescent probe into the brain of the investigated mice. **(C)** Representative fluorescent and optical overlay image of a brain with the fluorescent probe. Reproduced from Reference (Inagaki et al., 2019) under a Creative Commons Attribution 4.0 International License (https://creativecommons.org/licenses/by/4.0/).

An alternative and interesting approach was developed by S. Inagak and co-workers, who were able to monitor the brain activity in freely moving mice by using a luminescent probe (Figure 3). (Inagaki et al., 2019) They used a bioluminescence-based voltage indicator consisting of a voltage-sensing phosphatase combined with a fluorescent protein. An increase in the membrane voltage caused an enhancement of Förster resonance energy transfer between these two emitting units and, hence, a change in the ratio between the emission intensity of the two fluorescence probes. This allowed the authors to perform ratiometric measurements of brain activity by using a simple experimental setup (Figure 3A). Unfortunately, the ratiometric probe was working in the visible domain so the penetration of their technique in tissues was limited. They solved this problem by creating an optical window in the skull (i.e., hole covered by a glass lid) that enabled the luminescence of the probe to be recorded with a conventional fluorescence camera (Figure 3B). However, implanting an optical window presents several side effects such as pain, inflammation, and stress in the animal and, consequently, would alter the normal behaviour (including brain activity) of the analyzed mice.

## Monitoring Brain Activity Through Temperature: Fundamentals and Evidence

Brain temperature ( $T_{B}$ ) is the result of the energy balance between metabolism-induced heat delivery and heat dissipation mainly through thermal diffusion and blood perfusion (Kiyatkin, 2019). $T_{B}$ is calculated by solving Pennes bio-heat equation: (Pennes, 1948)

$$\rho c\frac{\partial T_{B}}{\partial t}=\frac{\partial }{\partial x}\left(k\frac{\partial T_{B}}{\partial x}\right)-\rho_{b}c_{b}\omega \left(T_{B}-T_{b}\right)+q$$ (1)

where ρ , c , k are the density, specific heat, and thermal conductivity of the brain tissue, $\rho_{b}$ , $c_{b}$ are the density and specific heat and thermal conductivity of blood, ω is the local blood perfusion rate, q is the local metabolic heat generation rate (a direct indicator of metabolic activity), and $T_{b}$ is the temperature of blood.

**FIGURE 4:**
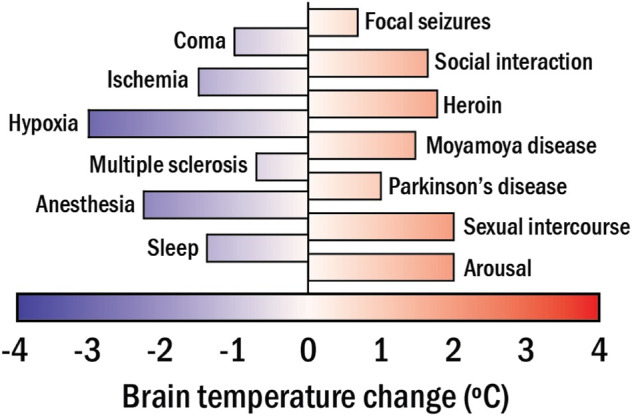
The range of brain temperature fluctuations detected in mammals in apparent response to different stimuli, activities, and diseases.

Eq. 1 reveals that any change in the metabolic activity of the brain has an impact on its temperature. Importantly, alterations of brain metabolic activity directly indicate changes of the brain functional activity (caused by the response to an external stimulus or by disease development affecting brain functions), owing to neurometabolic coupling. (Viswanathan and Freeman, 2007). The above observations suggest a new approach to measuring brain activity through its temperature. Extensive bibliography reports on significant changes in the brain temperature caused by alterations in the neuronal activity in response to different stimuli or diseases (see Figure 4). (Kiyatkin, 2019) Some representative examples are:

- The administration of psychoactive drugs (cocaine or heroin) can induce moderate (1.5°C) brain heating. (Bola and Kiyatkin, 2017). Larger heating (up to 5°C) can be achieved by administration of methamphetamine (Brown and Kiyatkin, 2005).
- Cardiac arrests in monkeys have been found to cause a brain temperature reduction of 1°C (Takeda et al., 2012).
- Social interaction has been found to have a limited but measurable impact on brain temperature (0.2°C) (Kiyatkin, 2007). For instance, interaction with humans can induce brain temperature increments in cats as large as 1°C (Delgado and Hanai, 1966).
- Neurodegenerative diseases (Parkinson’s) can induce brain temperature fluctuations up to ± 0.5°C (Sai et al., 2014; Chen et al., 2020).

It should be noted here that the unequivocal relation between brain temperature and brain activity could be established only when other parameters/conditions included in Eq. 1 are kept constant or their contribution is known and quantified. For instance, alterations in blood temperature or blood perfusion due to reasons different to changes in brain activity could induce changes in brain temperature too. Hence, there is a risk of an erroneous assignment of thermal changes to variations in brain activity.

When all this body of evidence is analyzed together, it is clear that brain temperature is an important physiological parameter, which fluctuates within the normal physiological continuum by measurable quantities (3–4°C). This makes specially desirable the development of technologies capable of measuring the brain temperature with a resolution well below 1°C. This is not an easy task. The brain is an organ thermally and mechanically isolated from the environment so the temperature of the scalp (easy to measure) and the brain itself can differ significantly. As a consequence, conventional infrared thermometry (fast and inexpensive) is not valid for brain temperature imaging as it cannot provide subcutaneous thermal readings (as in the case of the example shown in Figure 3). Instead, brain temperature has been measured so far using two technologies:

- *Intracranial implantation of thermal sensors* (thermocouples or optical fibres) (Musolino et al., 2019). This approach provides absolute and reliable thermal readouts without needing animal immobilization, but requiring skull piercing that, as mentioned earlier, is accompanied by several side effects. A representative example of this approach is the work published by R. Aaron Bola and co-workers who were able to monitor the impact of heroin administration in the temperature of brain in rats (Bola and Kiyatkin, 2017).
- *Diffusion-weighted imaging thermometry* (Odéen and Parker, 2019). In this case, the temperature of the brain is retrieved from the temperature of the cerebrospinal fluid (CSF), which is in turn assessed via the measurement of its temperature-dependent local diffusion coefficient. Like fMRI, this approach requires anaesthesia. Several examples of the potential application of fMRI for thermal imaging of the brain can be found in the review published by Huan Wang and co-workers (Wang et al., 2014).

The main limitations of these two approaches are their invasiveness and the requirement of anaesthesia, respectively. As explained above, these two factors alter brain activity making unbiased measurements of this parameter impossible. Technologies able to measure brain temperature in freely-moving and unharmed animals are necessary.

## Monitoring Brain Activity Through Temperature: Luminescence Thermometry

Nanostructures with temperature-dependent luminescence are referred as to luminescent nanothermometers (LNTs). (Brites et al., 2012; 2019). Visible-emitting LNTs were initially used for intracellular studies, providing excellent results, and motivating the community to implement their use in animal models. (Okabe et al., 2012; Maestro et al., 2013; Uchiyama et al., 2017; Okabe et al., 2018; Piñol et al., 2020; Sugimura et al., 2020). As commented above, tissues show a high extinction coefficient in the visible domain so that visible emitting LNTs have been found not appropriate for subcutaneous thermal sensing (del Rosal and Jaque, 2019).

**FIGURE 5:**
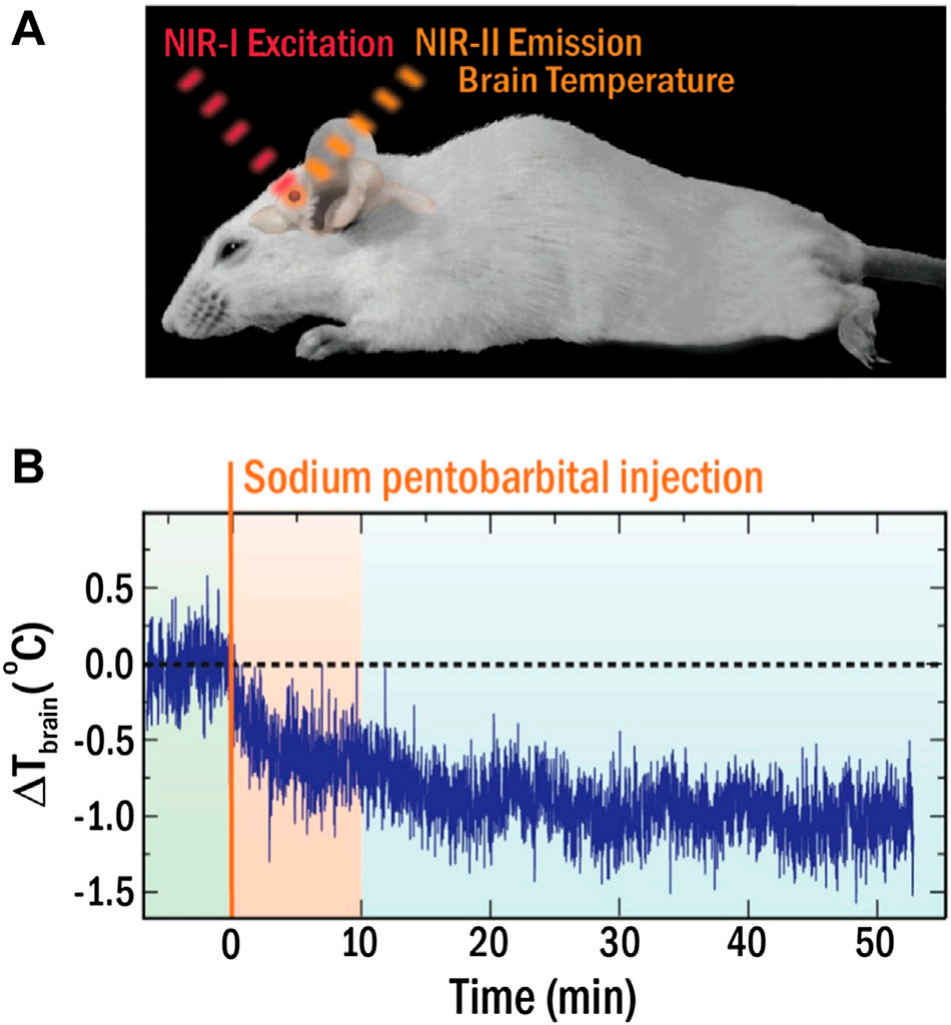
**(A)** Schematic representation of the experimental approach for *in vivo* thermal sensing in the brain with NIR-LNTs. **(B)** Time evolution of brain temperature during coma induction. Reproduced with permission from Reference (del Rosal et al., 2018).

This limitation forced the community to develop LNTs operating within the optical transparency windows of biological tissues, so called near infrared (NIR) spectral ranges (NIR-I 700–950 nm, NIR-II 1000–1350 nm) where light-tissue interaction is minimal (Smith et al., 2009; Golovynskyi et al., 2018; Song et al., 2018). Numerous LNTs operating in these windows (NIR-LNTs) have been recently developed as a consequence of an incessant research activity, including semiconductor- and lanthanide-based nanomaterials (Cortelletti et al., 2018; Nexha et al., 2021; Jiao et al., 2022). However, few of them have been applied *in vivo* (del Rosal et al., 2017). The spectral analysis of the radiation emitted by Ag_2_S-based NIR-LNTs has enabled intratumoral thermal sensing during photothermal treatments and *in vivo* detection of inflammation and damage in ischemic tissues (Ximendes et al., 2017; Santos et al., 2018). Recently, del Rosal and co-workers demonstrated how NIR-LNTs allow monitoring the brain cooling associated with the drastic reduction in brain activity caused by a pharmacological coma (see Figure 5). (del Rosal et al., 2018) In this case, the strong temperature dependence of the luminescence intensity of Ag_2_S nanoparticles (NPs) was used to assess the decrease in brain temperature after sodium pentobarbital injection (Figure 5B). The approach was simple: the real-time recording of the intensity of NIR radiation generated by Ag_2_S LNTs was used to obtain the time evolution of brain temperature. The experiments performed by del Rosal and co-workers showed clear advantages over previous approaches, including the use of non-ionizing radiation and a full remote nature. Yet, it has major limitations, as it required animal immobilization, anaesthesia, and skull piercing to introduce the NIR-LNTs into the brain. Furthermore, thermal reading was obtained from a simple analysis of the luminescence intensity, which is known to be affected by NPs diffusion and by tissue attenuation: an absolute readout of temperature becomes therefore impossible.

**FIGURE 6:**
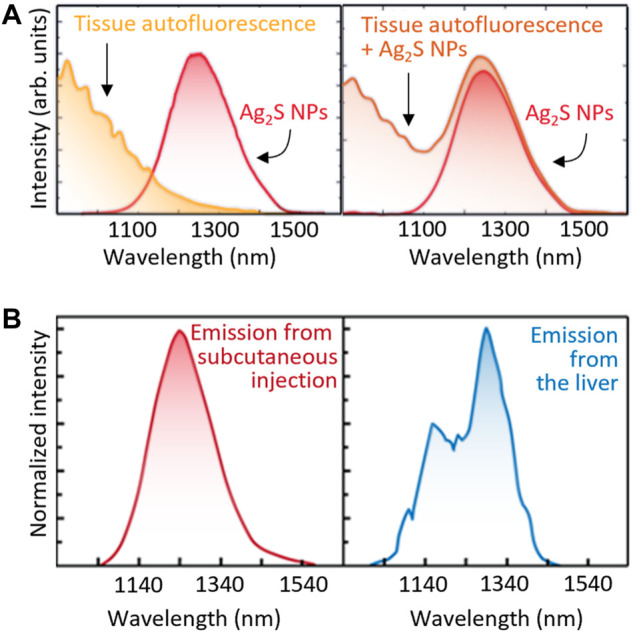
**(A)** Both the autofluorescence spectra of tissues and unperturbed emission band of Ag_2_S NPs (left) would contribute to the total emission spectra obtained in tissue (right). **(B)** Emission spectra of Ag_2_S NPs located subcutaneously and inside the liver. Relevant spectral distortion is evidenced in the case of Ag_2_S NPs are at the liver, that is, several millimetres under the skin.

The latter drawback could be overcome by using another luminescence property of the NIR-LNTs. Thermal reading (from many LNTs and from Ag_2_S NPs in particular) can be alternatively obtained from the analysis of the profile of the emission spectra. Initially, it was thought that the spectral features were not affected by the presence of tissues, thus the limitations of using the emitted intensity could be eliminated. Indeed, NIR-LNTs have been applied *in vivo* ignoring the non-negligible optical interferences. However, this has been recently demonstrated to be an over-simplification leading to false readouts when the spectral distortions due to tissues are not taken into account (Shen et al., 2020). These interferences have two causes:

- *Autofluorescence*. Although tissue autofluorescence was considered negligible in the NIR, this is not the case as most tissues show broad autofluorescence bands even in NIR-II (del Rosal et al., 2016). Thus, the emission spectrum recorded for an LNT within a tissue will overlap with the autofluorescence background (Figure 6A), leading to a distortion in the registered spectra that can be erroneously attributed to a temperature fluctuation.
- *Tissue extinction.* The large attenuation coefficient of tissues reduces the detectable luminescence of LNTs in *in vivo* experiments. Additionally, the wavelength-dependent nature of the tissues’ extinction spectra changes the shape of the emission band of the LNTs (Figure 6B). Recent results revealed how these tissue-induced distortions must not be neglected (Shen et al., 2020). If these distortions are attributed to thermal changes, they could lead to errors in temperature determination in excess of 10°C. This problem would also affect intracranial measurements as it has been widely reported that both brain tissues and skull also show strongly wavelength-dependent extinction coefficients (Golovynskyi et al., 2018).

**FIGURE 7:**
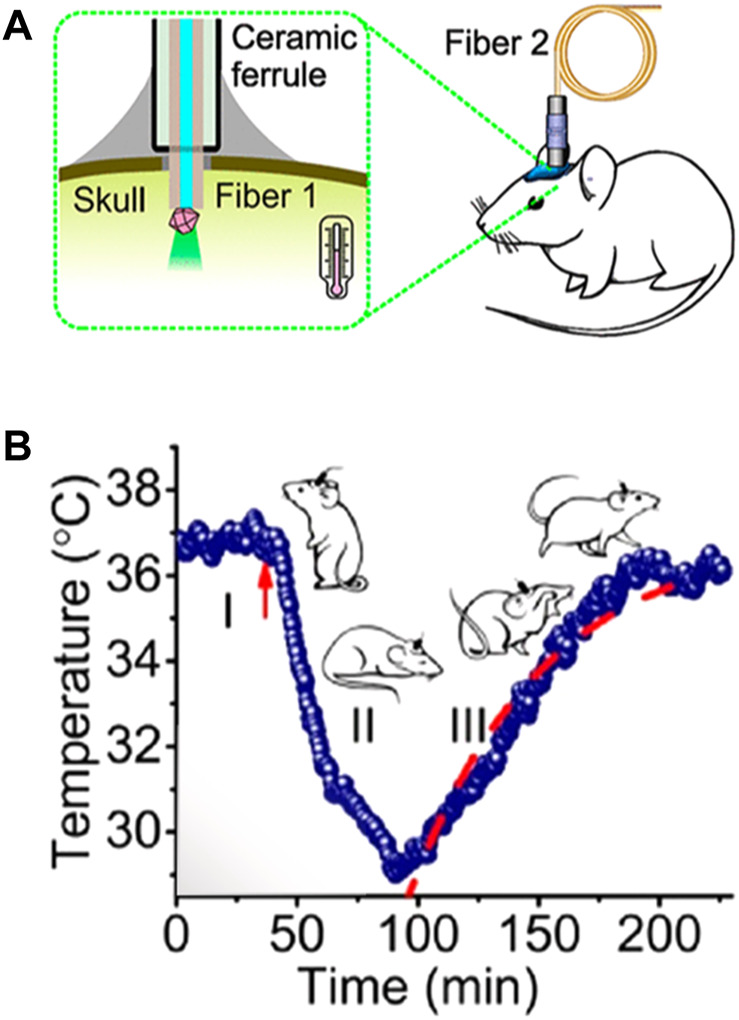
**(A)** Schematic representation of the experimental setup developed for the measurement of brain temperature by using a fluorescent microdiamond. **(B)** Time evolution of brain temperature during pharmacologically induced hypothermia process. Reproduced with permission from reference (Fedotov et al., 2020).

These problems are exclusively caused by the presence of biological tissue between the LNTs and the detection system. In absence of tissues, the spectral properties detected would be exactly those of the LNT and the thermal readout would be reliable. So, removing tissues between emitting LNTs and the optical detection system would solve this problem. An example of this alternative has been recently published by Fedotov and co-workers (Fedotov et al., 2020). In this case, the authors employed a diamond microcrystal with germanium-vacancy centres as a thermal sensor. Optical excitation and detection of the signal (luminescence) of the microdiamond was achieved by using an optical fibre that was mechanically coupled to the skull of the mouse (Figure 7A). The microdiamond was optically excited with green light and its luminescence was characterized by a narrow emission peak whose position around 600 nm is temperature-dependent. Based on this approach, the authors were able to monitor variations in brain temperature due to the reduction of brain activity caused by pharmacologically induced hypothermia (Figure 7B). The results obtained by Fedetov and co-workers were a nice proof of the potential of luminescence thermometry for both brain thermometry and brain activity monitoring but using an still invasive procedure.

An alternative route to avoid erroneous thermal readouts caused by tissue-induced distortions is to use lifetime-based LNTs (τ-LNTs). These are luminescent NPs with strongly temperature-dependent fluorescence lifetimes. In this case, remote thermal monitoring can be achieved from the analysis of the lifetime images. τ-LNTs have been extensively used for single cell studies. (Okabe et al., 2012; Hayashi et al., 2015; Uchiyama et al., 2017). Thermal sensing with τ-LNTs requires single wavelength measurements (spectrum acquisition is not necessary) so that it is not affected by tissue-induced spectral distortions. Although multiple scattering phenomena of photons within the tissue could possibly lead to distortions in the time decay curves—as different photons would travel different distances into the tissues—this phenomenon would be of importance only for very thick tissues and very short lifetimes. (Shen et al., 2022). Simple calculations demonstrate that the scattering-induced enlargement of lifetime for a tissue thickness of 1 cm the transit time of a photon is of the order of 10^−11^ s, which is several orders of magnitude smaller than the fluorescence lifetimes of τ-LNTs (ranging from 10^−8^ to 10^−3^ s, i.e., tens of nanoseconds to milliseconds).

Despite their great potential, τ-LNTs have been applied *in vivo* only for the detection of inflammatory processes by measuring the liver and subcutaneous temperatures using Ag_2_S NPs (Shen et al., 2022). Lanthanide-doped nanostructures and lanthanide complexes are an interesting alternative to the widely used semiconductor nanocrystals. They show long luminescence lifetimes and good stability in biological media. They have been even capable of absolute *in vivo* temperature sensing by lifetime thermometry (Tan et al., 2020). Despite their promising properties, lanthanide-based thermal sensors have not been yet applied to intracranial measurements. Such a demonstration is likely to occur in the next few years and it could constitute a revolution in the field.

These first demonstrations of *in vivo* lifetime-based luminescence thermometry are, indeed, promising and encouraging. Nevertheless, we should remark that intracranial thermal sensing would be much more challenging because of the high extinction of skull that would distort the laser pulse required for excitation and decrease the number of photons detected. Both limitations would be simultaneously solved by using bright luminescent NPs. This constitutes a major challenge since such an increment in the luminescent brightness should be achieved without affecting the lifetime-based thermal sensitivity.

## Delivering Nanoparticles into the Brain

One of the main drawbacks of luminescence thermometry is the necessity of introducing the luminescent contrast agents into the desired location within the body. In the case of the brain, they could be invasively introduced by intracranial injection, as mentioned above. To avoid that, it is desirable to deliver the NPs through the natural channels (circulatory system, cisterna magna) that connect the brain to the rest of the body. Another possibility to avoid skull piercing is introducing the NPs intranasally (Su et al., 2020). However, NPs would need to cross the blood brain barrier (BBB). This wall of cells protects the brain from infections and reduces the number of NPs that could be intracerebrally allocated. To avoid this issue, the surface of the NPs can be modified, or the permeability of the BBB controlled externally to open the door to the LNTs (Lombardo et al., 2020; Li et al., 2021).

This is one of the main challenges that needs to be tackled for intracranial luminescence thermometry to be a real alternative as a remote and less invasive brain activity sensing technique that avoids immobilization of the subject.

## Conclusion and Perspectives

In summary, the experimental evidence provided to date shows that the monitoring of brain activity requires minimally invasive techniques that do not require immobilization. Conventional techniques such as magnetic resonance or nuclear medicine have failed to meet this requirement and it has become necessary to explore new possibilities.

Brain temperature appears to be a physiological parameter whose unequivocal relationship with brain activity is widely demonstrated. However, the characteristics of the brain and the fact that it is mechanically isolated from the environment make measuring its temperature non-invasively a challenge of great technical difficulty. This paper describes pioneering results that demonstrate how the use of luminescent thermometers can constitute a new way to overcome this challenge. Although promising, these results are still premature and not only show that there is a way, but also show that this path will not be without obstacles. These difficulties define the different lines along which the scientific community should work to be capable of using luminescence thermometry as a brain activity sensing technique in the future:

i) It is mandatory to demonstrate the reliability of thermal measurements provided by luminescent thermometers. For this it is necessary to precisely know the effect of the extinction of tissues in the spectroscopic parameters used for thermal reading. The development of advanced data analysis techniques (including deep learning or the use of neural networks) should be developed to compensate for these effects and achieve reliable readings.
ii) It is necessary to identify new thermal probes whose operation is based on spectroscopic parameters that are not affected by the extinction of tissues, such as fluorescence lifetime. The application of these novel nanothermometers will require the development of new experimental systems. The use of pulsed lasers as excitation sources and point detectors with low response times will most likely be necessary. In addition, it is essential to develop theoretical models and calculation platforms that allow the thermal images obtained to be interpreted properly to correlate them with changes in brain activity. These must consider not only the thermal characteristics of brain tissues and the bone structure that protects them, but also the influence of changes in metabolic activity and blood flow that occur in animal models.
iii) The development of novel structures for brain thermometry should be accompanied by systematic studies on the possible adverse (toxic) effects of such nanostructures when incorporated into the brain. *In vitro* studies are of course required, but not sufficient. The impact of luminescent nanoparticles within the brain on the metabolic activity and on the general behavior of individuals must be also examined. Problems related to nanoparticle diffusion within the brain and nanoparticle decomposition must also be thoroughly studied before any potential clinical application.
iv) Finally, the need of performing all these measurements on freely moving animals will require the re-design of experimental animal imaging systems. Fluorescence-based thermal measurements of brain should be accompanied by a continuous tracking of animal activity. These optical systems should also be adapted to metabolic cages.

We are firmly convinced that by following the indications described, in the next years we will witness the appearance of new pre-clinical devices capable of measuring brain activity in freely moving animals using luminescent thermometry.

The ability of measuring brain temperature will also open new paths in pre-clinical research. For instance, knowing brain temperature in real time will allow us to modify it in a controlled way by, for instance, the use of infrared lasers. This, in turn, will make it possible to control neuronal activity by thermal treatments. Precise knowledge of brain temperature will also allow to study how this parameter affects the permeability of the brain blood barrier and to develop therapies that will take advantage of temperature to increase the brain targeting efficiency of drugs and nanoparticles.
